# Alterations of perfusion and functional connectivity of the cingulate motor area are associated with psychomotor retardation in major depressive disorder

**DOI:** 10.1007/s00406-024-01896-8

**Published:** 2024-09-19

**Authors:** Tobias Bracht, Nicolas Mertse, Sigrid Breit, Andrea Federspiel, Roland Wiest, Leila M. Soravia, Sebastian Walther, Niklaus Denier

**Affiliations:** 1 Translational Research Center, University Hospital of Psychiatry and Psychotherapy, University of Bern, Murtenstrasse 21, Bern, 3008 Switzerland; 2 Translational Imaging Center (TIC), Swiss Institute for Translational and Entrepreneurial Medicine, Bern, Switzerland; 3 Institute of Diagnostic and Interventional Neuroradiology, University of Bern, Bern, Switzerland; 4 Department of Psychiatry, Psychosomatics and Psychotherapy, Center of Mental Health, University Hospital of Würzburg, Würzburg, Germany

**Keywords:** Depression, Psychomotor retardation, Arterial spin labelling, Functional MRI

## Abstract

Psychomotor retardation, characterized by slowing of speech, thoughts, and a decrease of movements, is frequent in patients with major depressive disorder (MDD). However, its neurobiological correlates are still poorly understood. This study aimed to explore if cerebral blood flow (CBF) and resting state functional connectivity (rs-FC) of the motor network are altered in patients with MDD and if these changes are associated with psychomotor retardation. Thirty-six right-handed patients with depression and 19 right-handed healthy controls (HC) that did not differ regarding age and sex underwent arterial spin labelling (ASL) and resting-state functional MRI (rs-fMRI) scans. Psychomotor retardation was assessed with the motoric items of the core assessment of psychomotor change (CORE) questionnaire. Patients with MDD had more pronounced psychomotor retardation scores than HC. Patients with MDD had reduced CBF in bilateral cingulate motor area (CMA) and increased resting-state functional connectivity (rs-FC) between the cluster in the CMA and a cluster localized in bilateral supplementary motor areas (SMA). Furthermore, increased rs-FC between the CMA and the left SMA was associated with more pronounced psychomotor retardation. Our results suggest that reduced perfusion of the CMA and increased rs-FC between the CMA and the SMA are associated with psychomotor retardation in patients with depression.

## Introduction

Psychomotor disturbances are frequent in major depressive disorder (MDD). The Diagnostic and Statistical Manual (DSM-5) lists the following items as related to psychomotor disturbances: psychomotor retardation/ agitation and loss of interest in previously enjoyed activities. Psychomotor retardation is more frequent than psychomotor agitation [1, 2]. It is characterized by a slowing of speech, thoughts, and a decrease of movements [3]. Psychomotor retardation including decreased physical activity has been repeatedly reported in patients with MDD and even in patients with remitted depression [1, 4]. Psychomotor retardation is particularly pronounced in melancholic depression [5, 6], a subtype of depression with more marked biological alterations [7, 8] and presumably greater reduction in symptom severity following pharmacological antidepressive treatment [9]. Furthermore, psychomotor retardation may predict treatment response to electroconvulsive therapy (ECT) [10]. Although psychomotor retardation is frequently encountered in depression, few studies have targeted the behavioral assessment or neurobiological underpinnings of this phenomenon. Studies using wrist actigraphy reported links to daily activities and symptom ratings [11]. Furthermore, reduced physical activity in subjects with MDD was associated with alterations in cerebral resting perfusion or white matter pathways in core components of the motor system, particularly in premotor areas such as cingulate motor area (CMA) and supplementary motor area (SMA) [12–17].

Motor behaviour underlies a network that primarily comprises the basal ganglia, the cerebellum, the primary and the supplementary motor cortex (SMA) [18–20]. Further regions that are essential for motivated and goal directed behaviour are the prefrontal cortex (PFC) and the anterior cingulate cortex (ACC). Both the PFC and the ACC have strong links to limbic brain regions (e.g. amygdala, hippocampus) [21, 22]. The ACC plays an important role for action-outcome learning, specifically the CMA integrates reward outcome information, action information and it contains projections to premotor cortical areas [21, 22]. Structural and functional alterations in PFC, ACC, CMA and SMA have been reported repeatedly in patients with depression [7, 13, 14, 23]. Functionally, these disturbances may lead to lack of motivation and drive consequently resulting in psychomotor retardation [12, 15, 24, 25].

Resting-state cerebral blood flow offers insights on locally altered states of brain metabolism [26]. Brain areas with tonically altered neural activity are expected to exert distinct patterns of functional connectivity with other brain areas [27]. It was the aim of this analysis to extend knowledge on the neurobiology of psychomotor retardation in MDD using a multimodal functional magnetic resonance imaging (fMRI) approach. First, we aimed to investigate if there are alterations of cerebral blood flow (CBF) in the motor network in patients with MDD. We hypothesized reduced CBF in motor areas in patients with MDD, as psychomotor retardation is a frequent sign in MDD. Second, we aimed to explore, if these alterations in CBF are associated with changes in resting-state functional connectivity (rs-FC) between areas of reduced CBF and regions within the motor network. Third, we hypothesized that more pronounced changes in CBF and in rs-FC within the motor network are associated with higher ratings of psychomotor retardation.

## Methods

### Participants

Thirty-six currently depressed individuals were recruited at the University Hospital of Psychiatry and Psychotherapy in Bern, Switzerland. Participants of the present study have also been included in previous reports [7, 23, 28–31]. Inclusion criteria for this study were a diagnosis of major depressive disorder (MDD) according to the Diagnostic and Statistical Manual of Mental Disorders (DSM-5) [32], a completed assessment of the core assessment of psychomotor change (CORE) questionnaire [33], age between 18 and 65 years and right-handedness as assessed using the Edinburgh Handedness Inventory [34]. We used the Mini International Neuropsychiatric Interview (MINI) to screen for psychiatric comorbidities [35] and the structured clinical interview for DSM-IV Axis II (SCID-II) to screen for personality disorders [36], which were exclusion criteria to participate in the study. Furthermore, we excluded participants with neurological disorders and with contraindications to perform an MRI-scan (e.g. claustrophobia). We used the 21-item Hamilton rating scale for depression (HAMD) [37] and the 21-item self-report Beck Depression Inventory (BDI-II) [38] to assess depression severity. To assess psychomotor retardation, we used a sum score of the items 3 (postural slumping), 10 (body immobility (amount, not speed)), 13 slowed movement (speed, not amount) and 15 (delay in motor activity), which comprises all motoric CORE-items besides those that are related to language. Out of the 36 patients 32 were on antidepressive medication (selective serotonin reuptake inhibitors (SSRI): *n* = 7; serotonin noradrenaline reuptake inhibitors (SNRI): *n* = 13; bupropion: *n* = 3; tricyclic antidepressants: *n* = 8; tranylcypromine: *n* = 1). Augmentation strategies were applied in 17 patients, thirteen with lithium and four with atypical antipsychotics. Furthermore, two patients took zolpidem for sleep induction.

We included 19 right-handed healthy controls (HC). Assessment for HC was identical to the patient group. Inclusion criteria were the absence of any present or past psychiatric disorder as assessed with the MINI. All participants provided written informed consent. The study was approved by the local Ethics Committee of Bern (KEK Bern; BASEC-number: 2017 − 00731).

### MRI acquisition

We acquired structural and functional brain data with a 3 Tesla MRI scanner (Magnetom Prisma, Siemens, Erlangen, Germany) using a 64-channel head and neck coil at the Swiss Institute for Translational and Entrepreneurial Medicine (SITEM) in Bern.

For acquiring anatomical data, we used a 3D T1-weighted MP2RAGE sequence with the following parameters: FOV = 256 × 256 mm^2^, matrix = 256 × 256, slices = 256, voxel resolution = 1 × 1 × 1 mm^3^, TR/TE = 5000/2.98 ms, TI = 700 ms and T2 = 2500 ms.

For calculating cerebral blood flow (CBF) we acquired data using a pulsed arterial spin labeling (PASL) sequence with PICORE Q2TIPS technique [39, 40]. Basic parameters for the PASL sequence were the following: FOV = 230 × 230 mm^2^, matrix = 64 × 64, slices = 22, voxel resolution = 3.6 × 3.6 × 6.0 mm^3^, TR/TE = 3300/13 ms, flip angle = 90° and PICORE Q2T perfusion mode. In total, we acquired 90 pairs of label/control volumes in the axial direction with a tagging bolus duration (TI1) of 700 ms saturation and an inversion time (TI2) of 2200 ms, whereas during TI2, the labeled blood perfuses the brain tissues, resulting in a decrease of the MR signal.

We acquired an 8-minute continuous resting-state BOLD fMRI scan with the condition ‘eyes closed’ to measure functional connectivity between brain areas. Image acquisition based on echo planar imaging (EPI) with the following parameters: 480 volumes with 48 slices per volume, FOV = 230 × 230 mm^2^, matrix = 94 × 94, voxel resolution = 2.4 × 2.4 × 2.4 mm^3^, TR = 1000 ms, and TE = 30 ms.

For computation of a field map, we acquired a magnitude and phase images with a double-echo spoiled gradient echo sequence with the following parameters: TR = 500 ms, TE = 4.92/7.38 ms, voxel resolution = 2.4 × 2.4 × 2.4 mm^3^, flip angle = 60°.

### Data analyses

#### Calculation of cerebral blood flow (CBF)

For calculation of cortical cerebral blood flow ($\:\left[\text{C}\text{B}\text{F}\right]=\frac{\text{m}\text{l}}{100\text{g}\:\text{m}\text{i}\text{n}}$), we analysed PASL data (180 label and control images) using in house MATLAB scripts (MATLAB R2023a, MathWorks, USA). Initially, we corrected the raw PASL volumes to minimize the impact of movement artifacts. Subsequently, we applied a field map correction to all the realigned volumes. Finally, we computed the CBF time series using the following formula: $\:\text{C}\text{B}\text{F}=\:\frac{\varDelta\:\text{M}}{2{\text{M}}_{0}\text{T}\text{I}1}\bullet\:{\text{e}}^{\frac{\text{T}\text{I}2}{\text{T}\text{i}\text{b}}}$, where $\:\varDelta\:\text{M}$ is the difference signal (control – labelled), $\:{\text{M}}_{0}$ the equilibrium brain tissue magnetization and $\:\text{T}\text{i}\text{b}=1650\:\text{m}\text{s}$ the decay time for labelled blood at 3 Tesla [41]. For improvement of signal-to-noise ratio, we calculated the average CBF for the time series. We computed the average CBF for the time series to improve the signal-to-noise ratio. Then, we co-registered these averaged maps to structural T1-weighted images and isolated CBF within grey matter using binary masks derived using the DL+DiReCT (individual space, AsegAtlas) [42, 43]. Finally, we used SPM12 software (http://www.fil.ion.ucl.ac.uk/spm) to normalize individual grey matter CBF to standard Montreal Neurological Institute space. Normalized volumes were smoothed with a 6-mm full-width at half maximum (FWHM) kernel to reduce partial volume effects. A whole-brain two-sample t-test was performed to compare regional CBF between groups. Age and sex were included as covariates, cluster forming threshold was set to p-value < 0.001 and to identify significant clusters we used a family wise error (FWE) correction of p-value < 0.05.

#### Calculation of functional connectivity (FC)

We analysed resting-state fMRI using the CONN 21a toolbox [44]. Pre-processing steps included the realignment and co-registration of EPI volumes to T1-weighted MP2RAGE, segmentation, and normalization to MNI space, and smoothing using an FWHM kernel of 8 mm. We applied band-pass filtering (0.008–0.09 Hz) to remove physiological signals and regress nuisance variables of each 5-time series within segmented white matter and cerebrospinal fluid and of 12 realignment parameters. Scrubbing outlier volumes with global BOLD signal or framewise displacement (FD) higher than the 95th percentile was performed using the Artefact Detection Tools (ART) toolbox implemented in CONN. For every subject, we averaged FD and DVARS, which is the spatial root mean square of the BOLD signal after temporal differencing [45].

We used a-posteriori the binary mask of the significant cluster of the CBF group differences for computation of seed-based rs-FC maps (first-level). Second-level between group analyses of the FC-maps were corrected with regressors age, sex, mean FD, and mean DVARS. We used a voxel threshold of *p* < 0.01 and a cluster correction with a false discovery rate (FDR) correction of *p* < 0.05.

### Correlational analyses

Within patients, we performed for CBF maps and for seed-based (seed: a-posteriori binary mask) rs-FC maps whole-brain linear regression analysis with the psychomotor retardation sum score of the items 3, 10, 13 and 15 of the CORE. We used a voxel threshold of *p* < 0.01 and a two-side cluster correction with a family wise error (FWE) correction of *p* < 0.05. CBF maps were corrected for age and sex, rs-FC-maps were corrected with regressors age, sex, mean FD and mean DVARS.

### Statistical analyses of clinical and demographic data

The Statistical Package for Social Sciences *SPSS 28.0* (SPSS, Inc., Chicago, Illinois) was used for analyses of clinical characteristics and demographics between patients and HC using independent t-tests or χ^2^ tests as appropriate for continuous and dichotomous data. To estimate the effect size of significant findings, we calculated Cohen’s d using the following formula: $\:d=\frac{T}{\sqrt{n}}$, where $\:T$ is the ratio of the difference in a number’s estimated value from its assumed value to its standard error in a significant cluster and $\:n$ is the number of subjects included in the analysis [46].

## Results

### Participant demographic and clinical characteristics

Patients and HC did not differ regarding age and sex (see Table 1 for clinical and demographic characteristics). Six patients met criteria for double-depression for many years (range 8 to 27 years).

**Table 1:** Demographics

	Patients	Controls	Statistics
Age (years)	43.8 ± 12.7	42.1 ± 13.4	*p* = 0.64
Sex (female/ male)	19/17	8/11	*p* = 0.57
Duration of current episode (months)	12.6 ± 10.5	N/A	N/A
Number of episodes	3.2 ± 2.1	N/A	N/A
HAMD-total	22.0 ± 5.6	0.7 ± 1	*p* < 0.001
BDI-total	27.6 ± 9.2	1.6 ± 2.4	*p* < 0.001
CORE-total	12.6 ± 7.8	0.2 ± 0.8	*p* < 0.001
CORE (psychomotor retardation)	3.6 ± 2.8	0.1 ± 0.3	*p* < 0.001

Abbreviations: N/A: not applicable

### Group differences in cerebral blood flow (CBF)

**Fig. 1:**
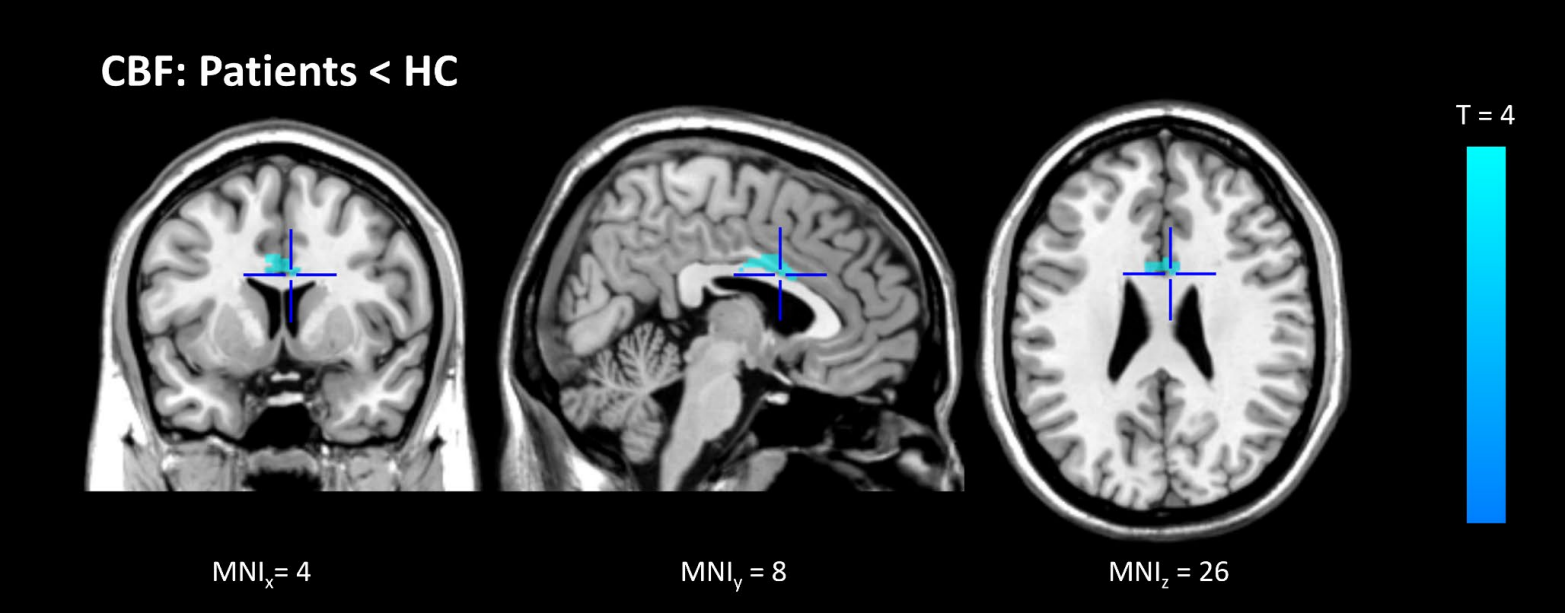
Group difference in regional CBF in grey matter in standardized MNI space Blue represents significant relative hypoperfusion in the bilateral CMA within patients

In the whole-brain group comparison, we found a large cluster in the bilateral cingulate cortex, mainly localized in the CMA, with lower CBF in patients compared to controls (MNI-coordinates (x, y, z): 4, 8, 26, cluster (number of voxels): 674, p-FWE: 0.009, T = 4.24, Cohen’s d = 0.57, medium effect size). See Fig. 1.

### Group differences in functional connectivity (FC)

**Fig. 2:**
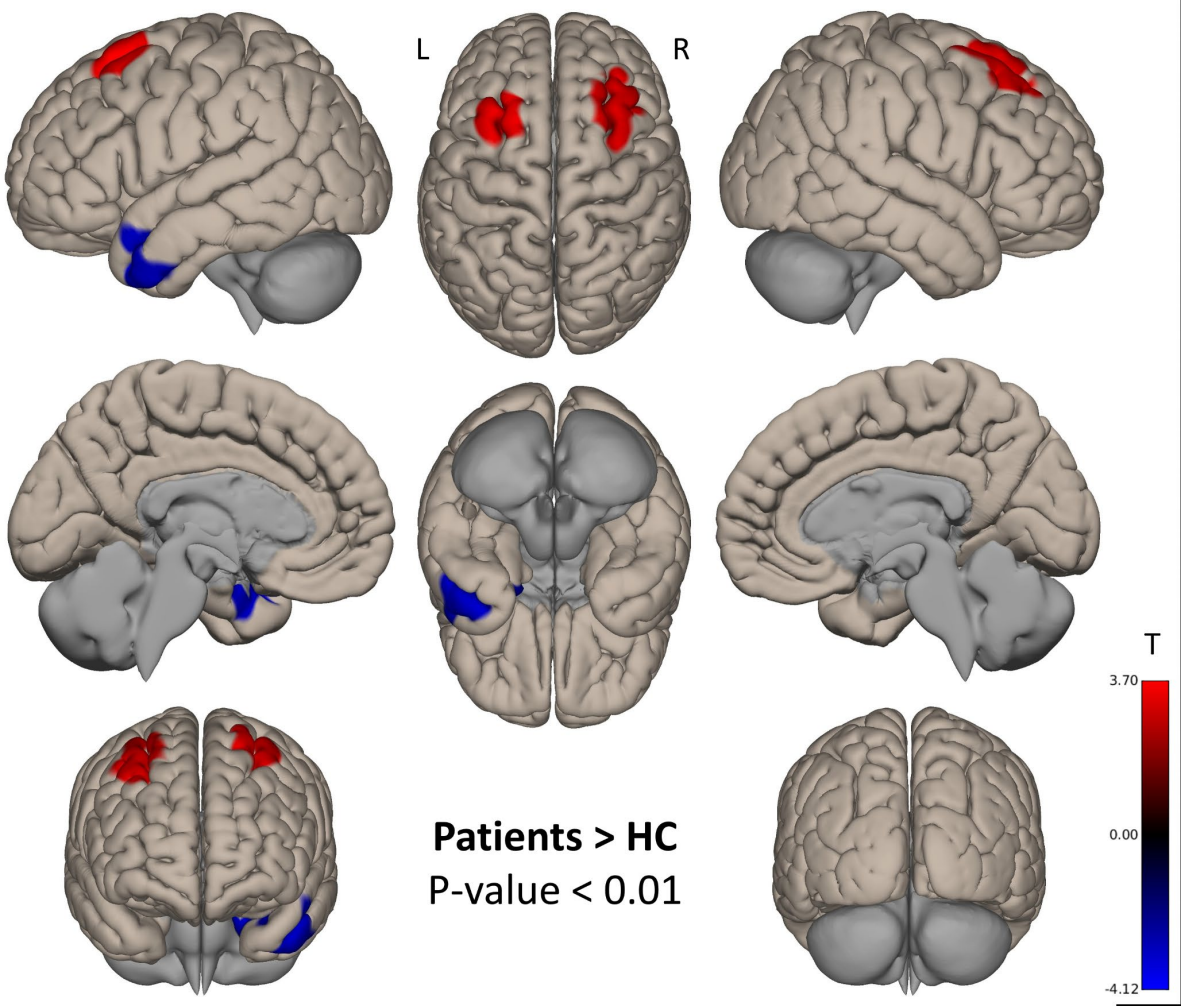
Group differences in seed-based rs-FC of the cingulate motor area Positive contrasts (patients > HC) are displayed in red, negative contrasts (patients < HC) are displayed in blue

After using the significant CBF cluster localized in the CMA as a binary seed mask, we found that patients had increased rs-FC in contrast to HC in bilateral superior and middle frontal gyrus (Cohen’s d = 0.50, medium effect size), localized in the lateral part of the SMA. In the opposite direction, HC showed increased rs-FC in the left temporal pole (Cohen’s d = 0.56, medium effect size). See Table 2; Fig. 2.

**Table 2:** Group difference in seed based rs-FC of the cingulate motor area

Comparison	Area	Hemisphere	Cluster (voxels)	MNI (x y z)	*p*-FWE
Patients > HC	Middle and superior frontal gyrus	R	503	32 40 48	0.000668
	Middle and superior frontal gyrus	L	474	-26 20 58	0.002082
Patients < HC	Temporal pole	L	728	-44 0 34	0.000052

Abbreviations: L: left hemisphere; R: right hemisphere

### Correlational analyses

**Fig. 3:**
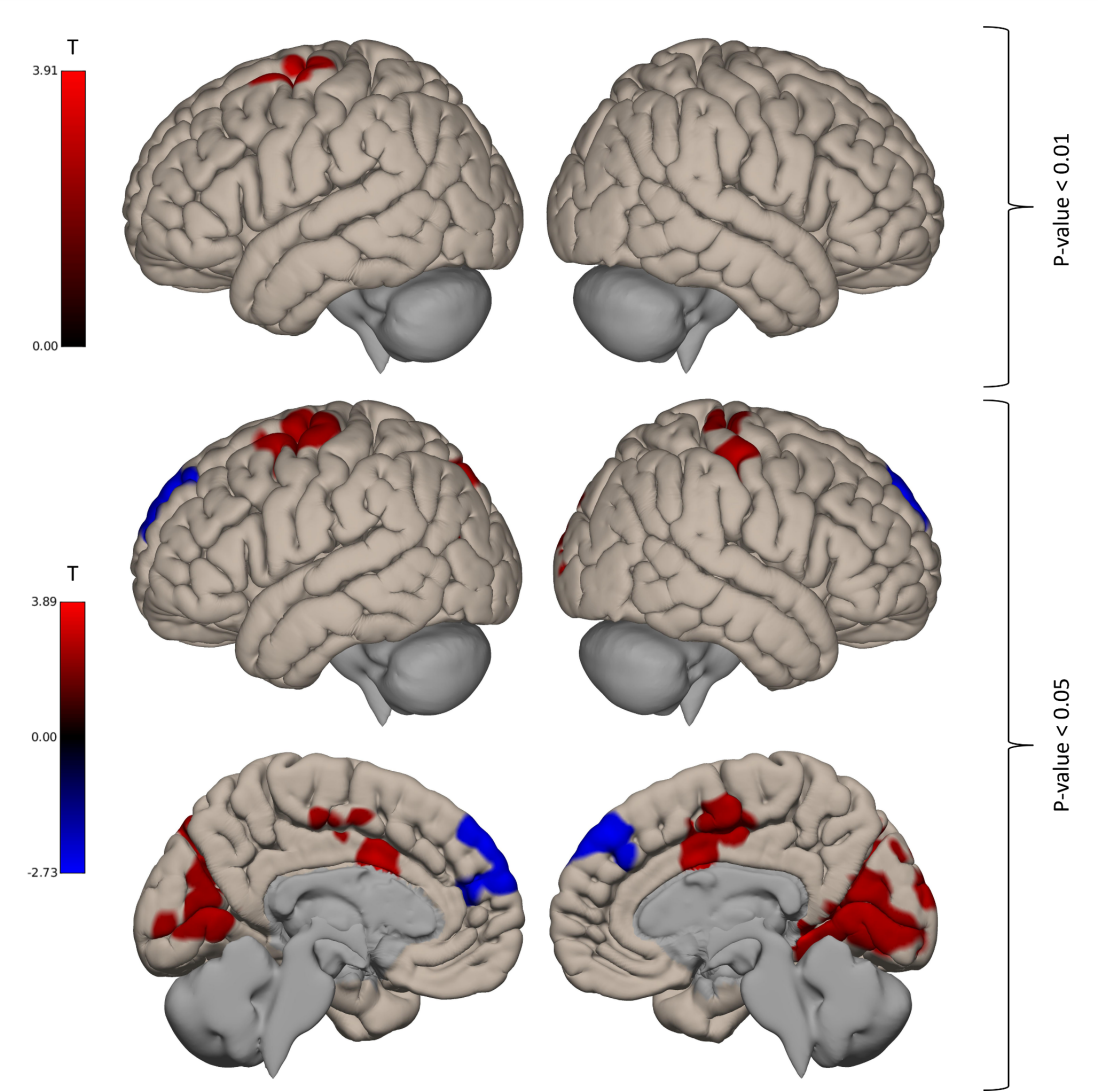
Correlation of psychomotor retardation and seed-based rs-FC of the CBF cluster within patients Positive correlations are displayed in red, negative correlations are displayed in blue. For visualization purpose, we also show results with a voxel threshold of *p* < 0.05

Within patients, we found a positive correlation between psychomotor retardation and rs-FC between the CMA (a-posteriori binary mask) and a cluster in the left hemisphere localised in the precentral gyrus, middle and superior frontal gyrus (MNI-coordinates (x, y, z): -26 -8 46, cluster (number of voxels): 455, p-FWE: 0.0031, Cohen’s d = 0.65, moderate effect size). No significant correlation was found for CBF and CORE motor retardation. See Fig. 3.

## Discussion

This study explored if alterations of local CBF are associated with rs-FC and psychomotor retardation in patients with MDD. In patients with MDD, we found reduced CBF in bilateral CMA, a segment of the ACC that is essential for the planning and initiation of movements. However, the severity of psychomotor retardation in patients failed to correlate with CMA CBF. Furthermore, there was an increase of rs-FC between the cluster with reduced CBF in the CMA and bilateral SMA in patients with depression. In addition, within patients increased rs-FC between the CMA-cluster and a cluster in the left SMA was associated with more pronounced psychomotor retardation.

Our results suggest a core role of the CMA, a segment of the ACC, for the pathophysiology of depression. The ACC integrates information from the limbic system, in particular the rostral ACC that receives input from the amygdala, the hippocampus, and the ventral striatum [47] regions that are heavily implicated in the neurobiology of depression. The CMA integrates information from the anterior and the posterior cingulum and projects to the SMA, a region that is essential for the initiation and inhibition of motor behaviour [21, 22, 48]. Our finding of reduced CBF in the CMA complements a previous ASL study pointing to a negative association between CBF in the ACC and apathy in depression [49]. Another study demonstrated a specific role of CMA perfusion for physical activity in MDD [14]. Psychomotor retardation in depression has also been associated with altered perfusion patterns in the insula, frontal brain regions, and the SMA [14, 16]. Overall, findings of increases and decreases of CBF in depression have been reported in widespread brain region and its functional relevance remains to be elucidated [50].

The lower CBF in the CMA indicates altered brain metabolism in this area, suggesting aberrant flow of information from and to this brain region. Indeed, in addition to our finding of reduced CBF in the CMA, we found increased rs-FC between the CMA and bilateral superior and middle frontal gyri, localized in the lateral parts of the SMA in patients with MDD. Increases of rs-FC between the CMA and the left SMA were associated with more pronounced psychomotor retardation in patients. Psychomotor retardation is thought to arise from multiple alterations within the cerebral motor network, including components such as CMA, SMA, primary motor cortex (M1), thalamus, cerebellum, and basal ganglia. In fact, prior unimodal work has shown alterations in these components or white matter pathways connecting them [1, 2, 12–15, 24]. In depression with psychomotor retardation rs-FC was increased between thalamus and M1/SMA, SMA and M1, as well as between thalamus and putamen [1]. In line with these connectivity patterns, the current study provides information on CBF and connectivity in the CMA. Results suggest that psychomotor retardation cannot be simply attributed to reduced perfusion in the CMA alone but rather to alterations of a connectivity within the motor circuitry. Aberrant coupling between the CMA and the SMA probably relates to lack of drive and reduced daily activity, features that are commonly observed in patients with depression [4, 11]. Neural activity in the motor circuit is altered in patients with psychomotor disturbance [51]. The net behavioral effect of retardation may arise from lack of activity in some components or from concurrent increases of activity within the circuit. The interactions between circuit components can have inhibitory and excitatory effects that are balanced within the system. In depression, we may assume that this balance is disturbed. However, the exact mechanism is yet to be unravelled. Still, research in schizophrenia with psychomotor slowing suggests some transdiagnostic commonalities, particularly relating to SMA and cerebellar connectivity [52–55]. Finally, the alterations in cortical motor areas and the dense connectivity within the motor circuit hold promise for non-invasive brain stimulation techniques to ameliorate retardation or slowing [1, 56] .

Finally, our study has some limitations. First, sample size is relatively small. Much larger sample sizes may be required to substantiate reproducible results [57]. However, we took great care to reduce the risk of false positive results by checking for outliers and by applying a stringent correction for multiple comparisons. The effect sizes (Cohen’s d > 0.5) also support the credibility of our findings. Second, most patients were medicated with antidepressants which may affect motor behaviour and measures of CBF, and rs-FC. Third, no objective measures of motor activity (e.g. actigraphy) were available allowing for assessment of spontaneous motor activity also outside the interview situation. Fourth, our finding of reduced CBF in the CMA is followed up by rs-FC analyses that are linked to psychomotor retardation. However, CBF reductions in the CMA did not correlate with psychomotor retardation. Therefore, this finding does not imply specificity regarding psychomotor retardation. Fifth, we chose a data driven approach (CBF reductions) to investigate functional networks associated with psychomotor retardation. Thus, identified networks related to psychomotor retardation are far from complete and further structures relevant to motor behaviour (e.g. basal ganglia, cerebellum) are not considered in our analyses [1, 2].

In conclusion, our analysis suggests that reduced perfusion of the CMA and increased rs-FC between the CMA and the SMA are associated with psychomotor retardation in patients with MDD. This finding is highly plausible, given the integrative role of the ACC for emotion processing and action planning and the frequently reported alterations of structure and function of the ACC in patients with depression [7, 58, 59]. Furthermore, the CMA has a critical role within the motor circuit.
